# Analysis of the Microbiome on the Surface of Corroded Titanium Dental Implants in Patients with Periimplantitis and Diode Laser Irradiation as an Aid in the Implant Prosthetic Treatment: An Ex Vivo Study

**DOI:** 10.3390/ma15175890

**Published:** 2022-08-26

**Authors:** Anna Wawrzyk, Mansur Rahnama, Weronika Sofińska-Chmiel, Sławomir Wilczyński, Beata Gutarowska, Adam Konka, Dagmara Zeljas, Michał Łobacz

**Affiliations:** 1 Silesian Park of Medical Technology Kardio-Med Silesia in Zabrze, M. Curie Skłodowskiej 10C Str., 41-800 Zabrze, Poland; 2 Chair and Department of Oral Surgery, Medical University of Lublin, Chodźki 6, 20-093 Lublin, Poland; 3 Analytical Laboratory, Institute of Chemical Sciences, Faculty of Chemistry, Maria Curie Skłodowska University, Maria Curie Skłodowska Sq. 2, 20-031 Lublin, Poland; 4 Department of Basic Biomedical Science, Faculty of Pharmaceutical Sciences in Sosnowiec, Medical University of Silesia, Kasztanowa 3, 41-205 Sosnowiec, Poland; 5 Department of Environmental Biotechnology, Faculty of Biotechnology and Food Sciences, Lodz University of Technology, Wólczańska 171/173, 90-530 Lodz, Poland; 6 Faculty of Drilling, Oil & Gas, AGH University of Science and Technology, Al. Mickiewicza 30, 30-059 Krakow, Poland

**Keywords:** titanium implant, oral microbiome, diode laser, peri-implantitis, irradiation

## Abstract

The paper presents the optimization of diode laser irradiation of corroded dental implants in order to reduce the number of microorganisms associated peri-implantitis. The research included the identification of microorganisms on the surface of removed dental implants in patients with peri-implantitis and the assessment of the biocidal effectiveness of the diode laser against these microorganisms. Laser desorption/mass spectrometry (MALDI-TOF MS) was used to identify microorganisms and metagens were examined by next generation sequencing (NGS). Irradiation was performed with a diode laser with a wavelength of λ = 810, operating mode: 25 W/15.000 Hz/10 μs, average = 3.84 W with the number of repetitions t = 2 × 15 s and t = 3 × 15 s. The structure and surface roughness of the implants were analysed before and after laser irradiation by optical profilometry and optical microscopy with confocal fixation. In total, 16 species of Gram-positive bacteria and 23 species of Gram-negative bacteria were identified on the surface of the implants. A total of 25 species of anaerobic bacteria and 12 species with corrosive potential were detected. After diode laser irradiation, the reduction in bacteria on the implants ranged from 88.85% to 100%, and the reduction in fungi from 87.75% to 96.77%. The reduction in microorganisms in the abutment was greater than in the endosseous fixture. The applied laser doses did not damage, but only cleaned the surface of the titanium implants. After 8 years of embedding, the removed titanium implant showed greater roughness than the 25-year-old implant, which was not exposed to direct influence of the oral cavity environment. The use of a diode laser in an optimised irradiation dose safely reduces the number of microorganisms identified on corroded dental implants in patients with peri-implantitis.

## 1. Introduction

Peri-implantitis is an inflammatory process that affects the tissues around the implant negatively and causes the supporting bone loss. The greatest risk of peri-implantitis is observed in the patients who do not care about cavity hygiene and are not regularly examined after implant treatment is completed. One of the causes of peri-implantitis can also be the excessive accumulation of microorganisms (including pathogens) on the surfaces of implantostructures [1,2].

The number of microorganisms settling on the implant surfaces depends on the surface structure of the biomaterials used. According to the European Society for Biomaterials, a biomaterial is any substance (unlike a drug) or a combination of substances of synthetic or natural origin that can be used at any time, in whole or in part to heal, enlarge or replace tissues of an organ, organ or organism [3]. There are metal, ceramic, polymer, carbon and composite biomaterials. Implants are medical components made of one or more biomaterials that when placed inside the body can remain for a long time [4].

Dental implants are exposed to degradation and various types of damage, e.g., pitting and freeting. Pitting results from the wear and tear of the material by means of cracks with which, for example, body fluids are pressed. It can be initiated by scratches formed in various stages of the implant production and exploitation of implants [3]. On the other hand, freeting occurs in the case of resting damages, in which the surfaces perform micro-vibrations in direct contact. The occurrence of such material damage is associated with the emission of corrosion products to the surrounding tissues and can cause cracks and damage the implant surface [4]. Microcracks can also occur in the case of material fatigue during the cyclic loading and unloading of the component [5].

In addition, the release of titanium debris into the surrounding tissues around the implant and the deposition of micro and nano particles in soft tissues can lead to inflammatory reactions and bone resorption [6,7].

One of the most promising long-lasting biomaterials used in dentistry is titanium and its alloys, which can stay in the body for over 25 years. Titanium and its alloys are characterised by the greatest biotolerance among the metallic biomaterials. According to research by Johansson and Han, bone tissue regeneration occurs better around the titanium-made implants than those made of titanium alloys [8,9]. However, any biomaterial in the environment of body fluids corrodes due to unfavourable conditions. The body fluids contain phosphates, chlorine, sodium, potassium, calcium and magnesium ions. In addition, high body temperature and the existing stresses promote corrosion. When an implant is inserted, the pH around it also changes. All these factors in a living organism create a very demanding environment for the implant, which not every biomaterial can withstand without damage [3]. 

One of the corrosive factors are also microorganisms capable of colonizing various metallic surfaces on dental implants in the form of biofilms, the so-called biofouling. *Fusobacterium* sp. (*F. nucleatum*), *Prevotella* sp. (P. denticolae), *Actinomyces* sp., *Porphyromonas* sp., *Veillonella* sp. and *Streptococcus* sp. comprise the bacteria colonizing dental implants the most frequently [10,11]. Among periodontopathogenic bacteria *Porphyromonas gingivalis*—one of the most important pathogens in chronic periodontitis, is capable of co-aggregation with *Fusobacterium nucleatum* and also with early colonisers such as *Streptococcus gordonii* [12], which could help explain its early appearance in the development of dental plaque biofilms [13]. Biofilm development is facilitated by the production of extracellular polymeric substances (EPS). Biofilms formed on the surfaces of metallic materials produce numerous metabolites, some of which are involved in electrochemical processes which can increase the possibility of corrosion caused by microbiological factors [14,15].

Titanium dental implants are exposed to the gradients of various chemicals concentrations in the oral cavity. These implants can be considered as electrochemical systems due to the differences in partial oxygen pressures in the supermucosal and submucosal environments and the gradients of numerous microbial metabolites [16,17]. Spatial oxygen gradients on the metal implant can lead to the formation of cathode half-cells and the generation of electric current. Derived from the field of microbial fuel cells, a metal electrode can provide electrons directly to bacteria in a biofilm (i.e., it acts as a biocathode), which can transfer electrons to oxygen, fumarate and iron compounds [18]. 

The corrosion resistance of titanium is related to the spontaneous formation of a layer of titanium oxide in contact with the oral cavity environment. The oxide is chemically stable, which protects titanium in contact with body fluids [19,20,21].

The titanium oxide (TiO_2_) layer protects the titanium surface against reacting with the electron acceptors such as oxygen. However, this layer is not acid-stable. 

There are numerous reports on the microbial corrosion of metals in the literature. The review by Li et al. presents a theory on microbial corrosion and shows the potential methods of its mitigation. However, in the clinical setting of dental implants, neither of these corrosion mitigation strategies are possible [17]. The occurrence of corrosion is clinically unfavourable and can shorten the exploitation implant in the body. The released metal ions can be toxic, causing pain and adverse biological reactions, which can even lead to implant rejection [3]. 

Titanium and its alloys are not characterised by good abrasion resistance, and the damaged surface of the implant reacts with the tissues and body fluids environment, intensifying corrosion, which results in the increasing roughness. The roughness of the implant surface and changes in its chemical composition due to microbial corrosion can affect the quantity and quality of dental plaque formation [22,23]. Surface roughness was identified as an important parameter regarding the ability of implant materials to anchor in the bone tissue. The greater implant roughness promotes the process of osseointegration, and, at the same time, favours colonization by microorganisms [24].

Microbial-mediated corrosion of titanium has been demonstrated in vitro in the presence of *Streptococcus mutans* [25]. *Streptococcus mutans* biofilm grown on a titanium surface and exposed to artificial saliva lowered the pH and it was observed that the oxide layer was thinner within 7 days of exposure. Within a much shorter time interval, titanium corrosion products were detected after just 90 min of exposure to *S. mutans*. [26]. 

The important stage in the peri-implantitis treatment is the removal of excessive amounts of plaque, using various cleaning methods, e.g., mechanical cleaning and surgical treatment. Modern methods of removing microorganisms do not increase the roughness parameters and do not cause microcracks on the implants surface. One such method is laser irradiation, which has become more and more common in dentistry for the decontamination of microbiologically contaminated surfaces. Very good results in the reduction in the number of microorganisms were achieved cleaning implants with a diode laser with a wavelength of 810 nm at the optimised doses [27,28].

Due to the fact that the biofilm also includes Gram-negative bacteria, which are more resistant to bacteriostatic and bactericidal components, their eradication by pharmacological methods is often ineffective. Therefore, alternative methods, including laser irradiation, are being sought.

Laser treatment decontaminates the surfaces covered with the biofilm [29]. The Er: YAG and Er, Cr: YSGG lasers, which did not effectively change the surface, proved to be effective in the decontamination of titanium surfaces contaminated with the biofilm in the oral cavity [30]. Wawrzyk et al. proved that diode lasers are biocidal effective and do not change the surface properties in optimised doses. At the same time, their use gives a measurable positive clinical effect. The exposed parts of the implant, such as the abutment and endosseous fixture, are most often irradiated. Their research confirmed that the effectiveness of laser irradiation depends on the type of laser surface. The exposed parts of the implant, such as the abutment and endosseous fixture, are most often irradiated [31].

The aim of the study was to present the microbiome on the surface of titanium dental implants used for 8 and 25 years and to identify microorganisms with a corrosive potential among them. Research was also carried out to optimise the doses of the diode laser with a wavelength of 810 nm, which can be used for the decontamination of microbiologically contaminated and corroded surfaces of abutments and endosseouse fixture during the peri-implantitis treatment. Research was also aimed at determining the structure and roughness of the implant surface before and after laser irradiation.

## 2. Materials and Methods

### 2.1. Patients and Materials

Two patients were selected for the study who reported to a dental surgeon with peri-implant inflammation. Peri-implantitis was found in both patients on the basis of radiological imaging showing a bone loss in the area of the implant neck, the reported pain and symptoms of inflammation around the implant and bleeding. Both patients were subjected to API (Aproximal Plaque Index) determination. The API index ranged from 45 to 80%, which indicates a lack of proper oral cavity hygiene. The PPD (Pocket Probing Dept) value was <4, which indicates the gingival pocket was slightly deepened. In the region of the implants, BoP (Bleeding on Probing) was positive, i.e., bleeding was detected during the examination. After the initial diagnosis, the decision was made to remove the implants and following this they were examined.

One implant was obtained from tooth 11 from a 90-year-old female patient with full-blown peri-implantitis (patient 1). She had the implant inserted 25 years earlier. In the clinical examination, the patient was diagnosed with an extensive inflammatory process manifested by redness and swelling of the tissues in the area of 11. The implant and the abutment, which were one of the pillars of the all-on-six work, were removed. Despite the disintegration of the implant with the bone tissue, it was still covered with soft tissues, confining the access to the factors of the oral cavity environment. 

The second implant was from a 67-year-old patient (patient 2) in position 44. The implant was placed in 2013 and removed in 2021. The patient had implants for 8 years. The removed implant was a pillar of the bridge in sections 44–46, supported by the implants in these positions. The reason for removing the implant was inflammation around the implant, which led to the loss of bone tissue and loss of implant stabilization in position 44. The process of bone loss caused by the implant was such that it was completely uncovered and exposed to the action of the oral cavity environment for 7 years.

### 2.2. Material for Microbiological Tests

The implants removed from the patients were rinsed in sterile saline and the suspension was used for microbiological tests. The cultured and multiplied microorganisms inhabiting the implants were frozen and stored in cryobanks at −80 °C. They were then revived and used to inoculate the implant to evaluate the biocidal efficacy.

The biocidal effectiveness of two variants of diode laser irradiation was tested on the removed dental implants used 8 and 25 years, which were inoculated around the cervix and endosseouse fixture, i.e., in the places most often exposed in peri-implantitis.

### 2.3. Material for the Surface Structure Study

The removed implants and the new implant were photographed for surface comparison. A detailed assessment of the structure and roughness was made before and after laser irradiation.

## 3. Methods

### 3.1. Photographs of Dental Implants

Photos of the removed titanium implants and the new implant were taken with a Canon EOS 750D camera, using a Canon 100 mm f/2.8 L EF Macro IS USM lens, exposure time of 1/200, f/25 shutter, 100 mm lens focal length and recorded in the CR2 format with 6000 × 4000 px resolution.

### 3.2. Microbiological Contamination of Titanium Implants

#### 3.2.1. Culture-Dependent Method

The implants were rinsed in the sterile saline. The suspension of the microorganisms was diluted 10-fold in sterile physiological saline. The suspension and dilutions were plated at 0.3 mL in 3 duplicates on the plates containing 5% Sheep Blood (Columbia Blood Agar, Oxoid, UK) and incubated in an aerobic atmosphere with 5% CO_2_ for 48 h at the temperature of 36 ± 2 °C. For fungi cultivation, inoculation was made on Sabouraud Agar (Oxoid, UK) and incubated for 5 days at 25 ± 2 °C. Anaerobic bacteria were detected by inoculation on the Scheadler Anaerobe Agar with horse blood (Oxoid, UK) and then incubated under anaerobic conditions at 36 ± 2 °C for 4 days. The rest of the fluid was used for metagenomic studies.

#### 3.2.2. MALDI TOF Method

Each colony grown on Columbia Blood Agar (Oxoid, UK) after 48 h of growing at 36 ± 2 °C under the aerobic conditions with 5% CO_2_ was isolated and identified using the matrix-assisted laser desorption/ionisation time-of-flight mass spectrometry (MALDI-TOF MS). This method was applied using IVD HCCA matrix (Bruker, Billerica, MA, USA). Microflex LT system (Bruker Daltonics, Bremen, Germany).

#### 3.2.3. Metagenomic Sequencing Method

For the comprehensive identification of the genetic material of all species of microorganisms inhabiting the titanium corroded implants, a metagenomic analysis was performed according to the principles described in Section Total DNA Extraction, Section Amplification of 16S rRNA Gene Fragment and Section Sequencing.

##### Total DNA Extraction

The resulting liquid sample (10 mL) was centrifuged for 10 min at 5900× *g*. After removal of the supernatant, the total DNA was extracted from the pellet using the Fast DNA Spin Kit for Faeces (MP Biomedicals, Illkirch, France) according to the manufacturer’s instructions. The DNA concentration was determined using a Qubit™ 2.0 fluorometer (Invitrogen, Carlsbad, CA, USA).

##### Amplification of 16S rRNA Gene Fragment

The bacterial 16S rRNA genes were amplified from the extracted total DNA using primers Bac341 and Bac805R at the concertation of 300 nM (Table 1). Each reaction contained KAPA HiFi HotStart DNA polymerase (Kapa Biosystems, Inc, Wilmington, MA, USA) at a concentration of 0.5 U. The matrix concentration of 10 ng and the 25 µL of the reaction volume were used. The PCR conditions included denaturation at 95 °C for 3 min, followed by 24 cycles of denaturation (98 °C for 20 s), hybridization (58 °C for 15 s), elongation (72 °C for 30 s) and an extension step at 72 °C for 1 min. Six prepared separate reactions were combined and purified using the Clean-Up Purification Kit (EURx, Gdańsk, Poland).

##### Sequencing

The sequencing libraries were constructed using the MiSeq Reagent Kit v3 (Illumina, San Diego, CA, USA). The libraries were sequenced in 2 × 300 bp paired-end mode using MiSeq by Eurofins Scientific (Germany).

### 3.3. Bioinformatic Analysis 

The quality control and adapter trimming were performed with fastp (version 0.19.5). Barcelona Supercomputing Center Plaça Eusebi Güell, 1-3 08034 Barcelona (Spain)The trimmed sequences were analysed using the Qiime2 software (2019.10 release). Then, the read sequences were shortened (forward/backward, 270/230), using the DADA2 plugin to obtain the amplicon sequence variants (ASV), filtering, denoising, combining the paired readings as well as removing chimeras were performed. The bacterial taxonomy was assigned to the ASV using the Naive Bayes classifier, based on the data from the Silva 132 SSU database, which included the V3-V4 region of the 16S rRNA gene, bound by the primer pairs Bakt_341F/Bakt_805R.

### 3.4. Laser Irradiation 

The Elexxion claros (Singen, Germany) laser with a fibre diameter of 600 µm at the wavelength of λ = 810 ± 10 nm was used for irradiation. The operating mode of the peri-implantitis surgical laser was tested: 25 W/15.000 Hz/10 µs, average = 3.84 W, applicator 600 µm, t = 2 × 15 s and t = 3 × 15 s with a 1 min break. All samples were irradiated with the sweep method.

The biocidal effectiveness of the laser was assessed using pathogens isolated from the removed implants, i.e., *Klebsiella oxytoca* (representative of Gram-negative bacteria), *Streptococcus constellatus* (representative of Gram-positive bacteria) and the yeast-like fungus *Candida guilliermondii.*

For comparison, laser irradiation was applied to the strains from the American Type Culture Collection (ATTC), i.e., *Staphylococcus aureus* ATCC 29213 (Gram-positive bacteria), *Escherichia coli* ATTC 25922 (Gram-negative bacteria) and the representative species of fungi was *Candida albicans* ATTC 10231.

An inoculum of the above-mentioned microorganisms with a densitometric value of about 106 CFU/mL (Colony Forming Unit) was applied to the abutments and endoessoues fixture of titanium implants (8 and 25 years old). Both parts of the implant were inoculated with 20 µL in 5 aliquots of 4 µL, allowing drying after each portion. The test was repeated three times. After irradiation, the implant was rinsed in sterile physiological saline. The control sample was not irradiated. Before and after the irradiation the microbiological tests of the samples were carried out in the same way as described in Section 3.2.1 this work.

### 3.5. Statistical Analysis

For the obtained results, mean values and standard deviations were calculated. The statistical significance of the differences for the reduction in the number of microorganisms after the laser irradiation was obtained in the least significant difference test (LSD) and ANOVA (one-way analysis of variance). The calculations were made with the Statistica 6.0 software (Statsoft, Tulsa, OK, USA) at the significance level (*p* < 0.05).

### 3.6. Analysis of the Surface Morphology of the New Titanium Implants and Those Withdrawn from the Oral Cavity

Two microscopic techniques described in Section 3.6.1 and the optical profilometry were used to determine the surface structure of the implants. The tests were carried out for the three implants: the new implant-control, the implant removed after 8 years and that removed after 25 years in the patients with peri-implantitis.

#### 3.6.1. Optical Microscopy

In order to visualise the surface in high resolution and contrast, the optical microscopy in the reflected polarised light technique was used with the Nikon Eclipse MA 200 metallographic microscope (Nikon, Tokyo, Japan). Additionally, to differentiate the inorganic and organic substances on the surface, the optical microscopy in the confocal mode using blue laser light with the wavelength of λ = 408 nm was used with the Nikon Eclipse MA 200 metallographic microscope (Nikon, Tokyo, Japan) with the C1 confocal attachment.

#### 3.6.2. Optical Profilometers

Optical profilometry enables the registration of three-dimensional images of the surface and the measurement of roughness parameters. This information makes a comprehensive assessment of the surface microgeometry of the tested material possible. The tests were carried out using the vertical scanning interferometry technique with the spectral range of coherent green light with a wavelength of λ = 515 nm on the Contour GT-K1 optical profilometer (Bruker-Veeco, Tucson, ZA, USA). Microgeometry maps and surface roughness measurements were determined for the scan sizes: 1261 × 946 µm, for the implant elements: endosseous fixture and abutment. Microgeometry maps were made for each element and roughness parameters were determined for the following areas: without laser irradiation and after laser irradiation with the dose of 2 × 15 s and the dose of 3 × 15 s. The roughness measurement (Ra) uncertainty was estimated taking into account repeatability, recovery, de-calibration of the apparatus and pattern uncertainty. The uncertainty was estimated for the two extreme points of the Ra measurement range (upper and lower limits) with the assumption of a trapezoidal distribution.

## 4. Results

### 4.1. Image of Dental Implants, Both New and Removed after 8 and 25 Years, Taken from Patients with Periimplantitis

**Figure 1:**
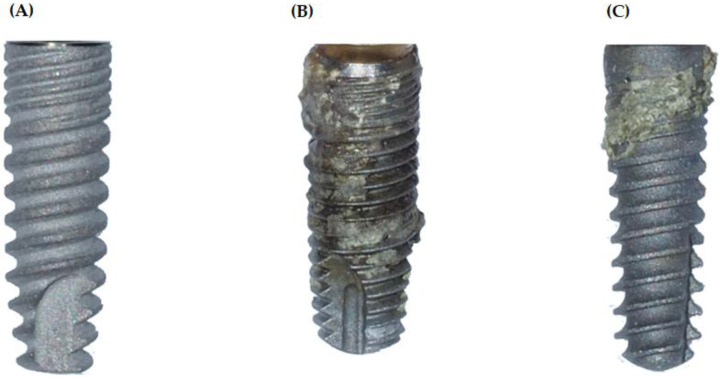
Dental titanium implants: new (**A**) and removed in the patients with full-blown peri-implantitis after 8 years (**B**) and 25 years (**C**).

In order to illustrate the changes in the surface structure of titanium implants over time and corrosion due to contact with the oral cavity environment, a photo was taken (Figure 1) showing that the new implant has the cleanest and smoothest surface. Slight traces of corrosion can be seen on the implant worn by patient 1 for 25 years, and the implant removed from patient 2 after 8 years of wear is very heavily corroded. The bone tissue remains are visible on both implants.

### 4.2. Microorganisms Isolated from the Surfaces of Dental Implants Used by Patients with Full-Blown Peri-implantitis Identified Using the MALDI TOF Technique

The cultured species of microorganisms isolated and identified by the MALDI TOF MS technique, living on the surface of implants used for 8 and 25 years by the patients with peri-implantitis, are presented in Table 2.

On the 8-year-old implant of patient 2, 12 species of bacteria and two species of fungi were identified by the MALDI TOF MS method. From the 25-year-old implant of patient 1, a slightly smaller number of bacterial species (8) and one species of fungus were identified. Streptococcus species were the most frequently recurring species in both patients. Among the microorganisms of patient 2 (25-year-old implant), 4 pathogens, and in patient 1 (8-year-old implant), three pathogenic species were identified. Most of the identified microorganisms belong to the natural flora of the oral cavity. Pyogenic Streptococcus conselatus, as well as Lactococcus lactis and Staphylococcus capitis, were isolated in both patients.

### 4.3. Metagenes on the Corroded Dental Implant

**Figure 2:**
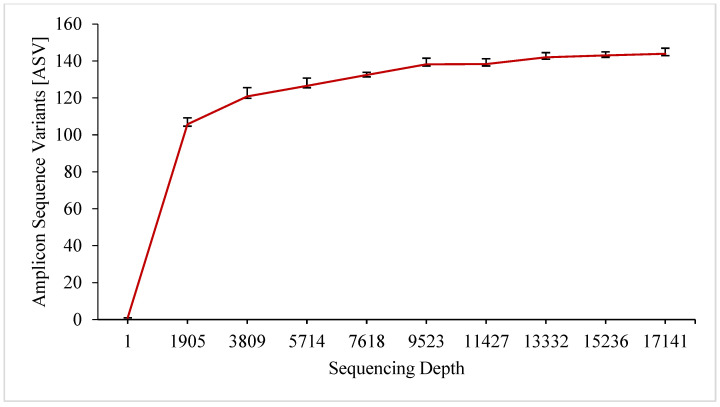
Rarefaction curve of microorganisms for the collective sample obtained from the 8- and 25-year-old implants.

The metagenomic studies allowed us to also identify the presence of genetic material in the species difficult to cultivate under the laboratory conditions. After V3-V4 sequencing, the 16S rRNA region 184,733 was obtained. After the quality control and assembly, 94,574 sequences were used to classify the bacterial taxonomy. The dilution curve reached a plateau at a depth of about 2000 readings, suggesting that a good representation of the microbial community was obtained for the analysed environment (Figure 2). 

**Figure 3:**
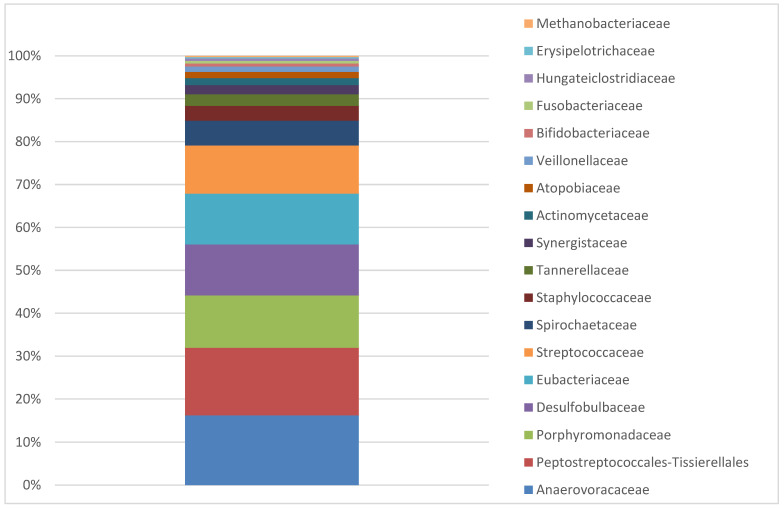
Abundance of bacterial families in the collective sample of implants removed from the patients with peri-implantitis after 8 and 25 years of use.

For the analysed sample, the alpha differentiation metrics were calculated, i.e., the Shannon differentiation index was 5.18475. For the sample of basic calibration of alpha differentiation, the Shannon differentiation index was equal to 5.18475. Figure 3 presents the percentage contribution of bacterial families identified on the dental implants of the patients with peri-implantitis (collective data for both cases, i.e., 8-year and 25-year-old implants).

Table 3 presents the species of microorganisms identified on the 8 and 25-year-old implants using the next-generation sequencing (NGS) method.

From the surface of implants removed in patients with full-blown peri-implantitis, 16 species of Gram-positive bacteria, 23 species of Gram-negative bacteria and 24 species of anaerobic bacteria were isolated and identified using the NGS method. Among the Gram-positive bacteria, Streptococcus (6) and Eubacterium (4) were the most numerous. Gram-negative microorganisms are most often bacteria of the genus Prevotella (8) and Treponema (4). Of the 39 identified species of microorganisms, 25 were classified as anaerobic. In the samples, the following pathogens were detected: *Dialister pneumosintes*, *Eubacterium nodatum*, *Fusobacterium nucleatum*, *Peptoniphilus lacrimalis*, *Porphyromonas gingivalis*, *Prevotella denticola*, *Prevotella nigrescens*, *Streptococcuema gordonii*, *Streptococcus mutans*, *Streptococcus dentoccus*, *Streptococcus mutans*, *Streptococcus dentoccus*, *Streptococcus mutans*, *Streptococcus dentoccus* and *Streptococcus mutans*. Among the identified microorganisms, 12 species of bacteria with corrosive potential were detected.

### 4.4. Reduction of Microorganisms on the 8 and 25 old Year Implants after Using 2 Dose Variants of Diode Laser 

All differences between the unirradiated and irradiated with laser samples in two variants were statistically significant (* in Table 4).

For the bacteria isolated from the implant surface, the reduction amounted to 89.83–100% (connector) and 88.85–100% (endosseous fixture), and for the fungi 88.05–100% (abutment), depending on the dose variant and surface area. The reduction in microorganisms on the 25-year-old implant is in every case greater than on the 8-year-old implant, with large visible traces of corrosion. Larger reductions were achieved with the Gram-negative than Gram-positive bacteria. Microorganisms isolated directly from the implant are more difficult to reduce than the ATTC standard strains. The use of three repetitions of 15 s each with a 1 min gap increased the reduction in the number of microorganisms for all tested microorganisms in relation to the variant of 2 × 15 s of irradiation. On both implants, the microorganisms diminished better on the surface of the abutments than on the endosseous fixture.

On the connector of the 25-year-old implant, the applied doses of laser irradiation eliminated the tested species of bacteria and fungi completely. On the endosseous fixture surface of the same implant, such an effect was not obtained for the bacteria irradiated with the dose of 2 × 15 s.

On the 8-year-old implant after laser irradiation, no microorganism isolated from the implants was eliminated in 100% from any surface. Such an effect was obtained only for Staphylococcus aureus ATTC on the connector in both irradiation variants and endosseous fixture in the 3 × 15 s variant.

The fungi reduced less than the bacteria. They were not completely eliminated from any endosseous fixture surface in any implant in both irradiation variants.

The smallest reduction in microorganisms was achieved on the 8-year-old implant in the 2 × 15 s irradiation variant for *Candida guillermondii*.

### 4.5. Analysis of the Implants Surface before and after the Laser Irradiation

#### 4.5.1. Optical and Confocal Microscopy

**Figure 4:**
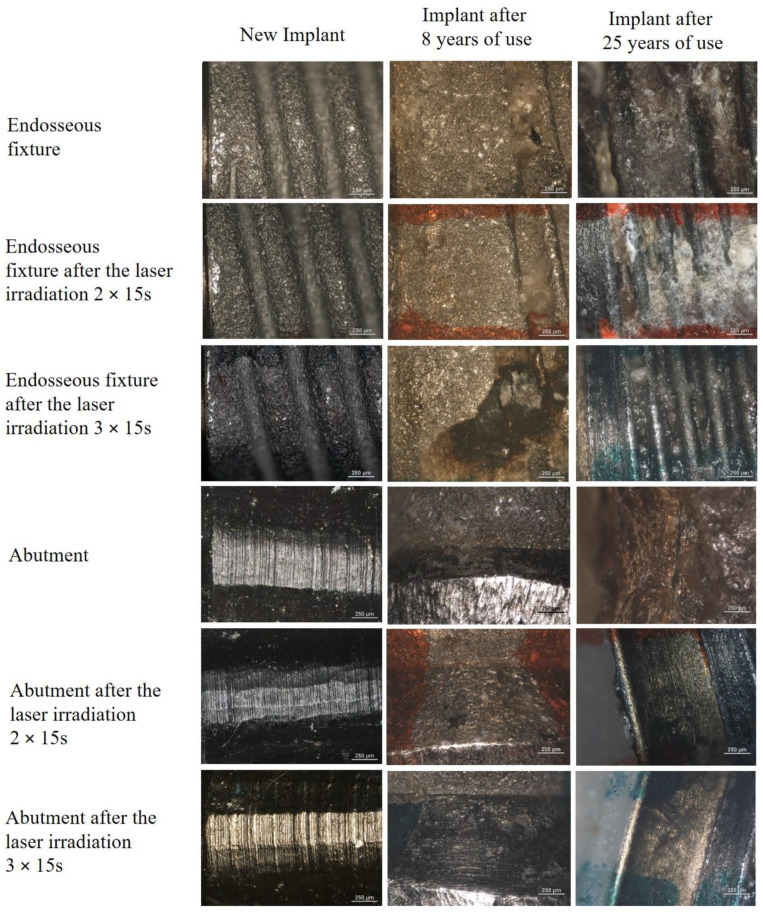
Reflected light microscopic images of the surfaces of the implants: (1750 × 1300 μm) new, removed after 8 and 25 years before and after the laser irradiation 25 W/15.000 Hz/10 μs, average = 3.84 W, during irradiation 2 × 15 s and 3 × 15 s.

In order to estimate the surface of new implants and those used by patients, microscopic images were taken using optical microscopy. The implants surface after the laser irradiation was also estimated. The test results are shown in Figure 4.

The research with the use of optical microscopy allowed for the observation of endosseous fixture surfaces and abutment before and after laser irradiation. The tests enabled the comparison of these surfaces in the new implant and those used for 8 and 25 years. The microscopic photos of the new endosseous fixture implant before and after laser irradiation showed no significant differences on the surface of the tested materials. A significant amount of contamination was observed on the endosseous fixture surface on the implants used by the patients. A greater accumulation of impurities was observed in the implant used for 8 years, which was manifested by the disappearance of characteristic cavities. The cleaning effect of the laser on the endosseous fixture surface was also observed in the case of applying the dose of 3 × 15 s, especially visible in the implant used for 25 years.

The microscopic photos of the new implant abutment show a smooth and homogeneous surface. The greatest amount of contamination was observed on the surface of the implant used for 25 years (bright areas on the microscopic images). On the surface of the implant used for 8 years, no areas smoothed after laser irradiation with the dose of 3 × 15 s were observed. In the case of the implant used for 25 years, abutment smoothing was observed after laser irradiation with the dose of 2 × 15 s and 3 × 15 s.

**Figure 5:**
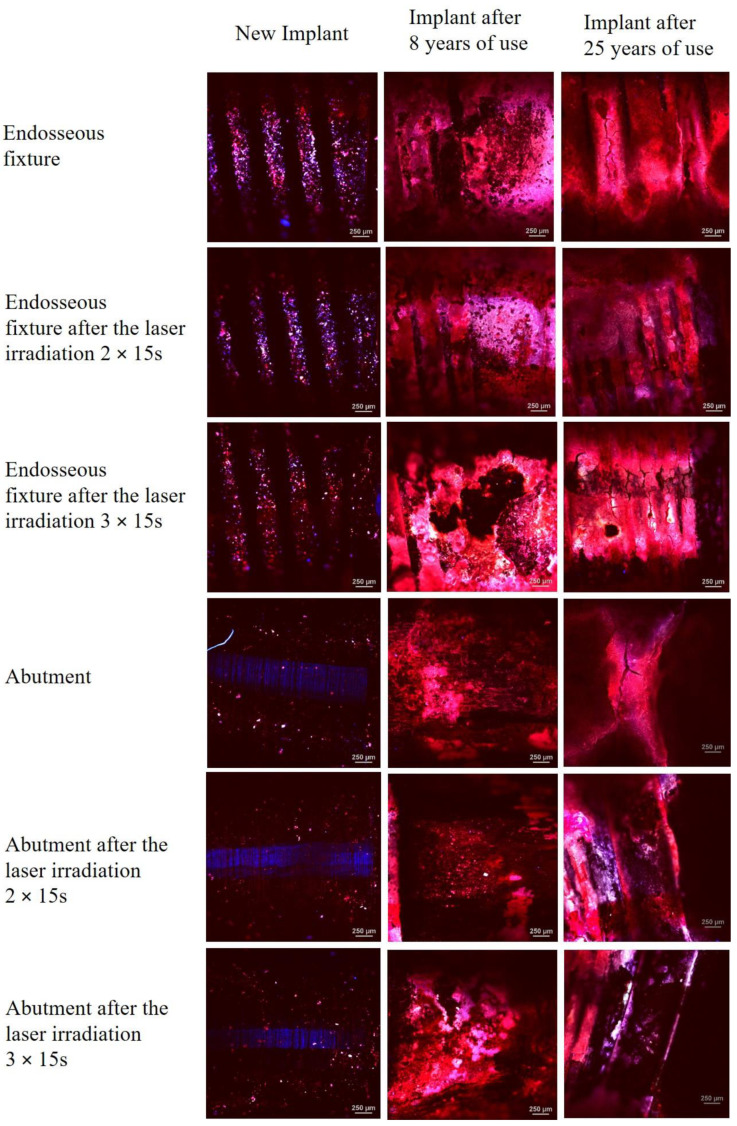
Microscopic images of titanium implant surfaces (2550 × 2550 μm) made in the polarised light for the implants: new, removed after 8 and 25 years, before and after the laser irradiation 25 W/15.000 Hz/10 μs, average = 3.84 W during the irradiation 2 × 15 s and 3 × 15 s Photographs taken under an optical microscope in the confocal mode.

Figure 5 shows the fluorescence images of new implants and those used by the patients using the confocal attachment with the violet laser of the wavelength of λ = 405 nm.

Microscopic examinations with the use of blue laser light confocal microscopy with the wavelength of λ = 408 nm allowed for the observation of endosseous fixture surface fluorescence and abutment before and after laser irradiation. The research was also aimed at comparing these surfaces in the new implant and those used for 8 and 25 years. The examination of the new implant showed a homogeneous surface of the endosseous fixture and abutment area. The microscopic images show a significant decrease in fluorescence on the endosseous fixture surface after laser irradiation with the doses of 2 × 15 s and 3 × 15 s. The area after the 3 × 15 s irradiation is characterised by the smallest fluorescence, which can indicate a smaller amount of organic phase on this surface. The largest areas of fluorescence can be observed on the endosseous fixture surface for the implants used for 8 and 25 years. Due to the surface structure, the endosseous fixture area favours the accumulation of the organic phase which is manifested by significant fluorescence. The abutment areas are characterised by much smaller fluorescence due to the much smoother surface.

#### 4.5.2. Optical Profilometry

**Figure 6:**
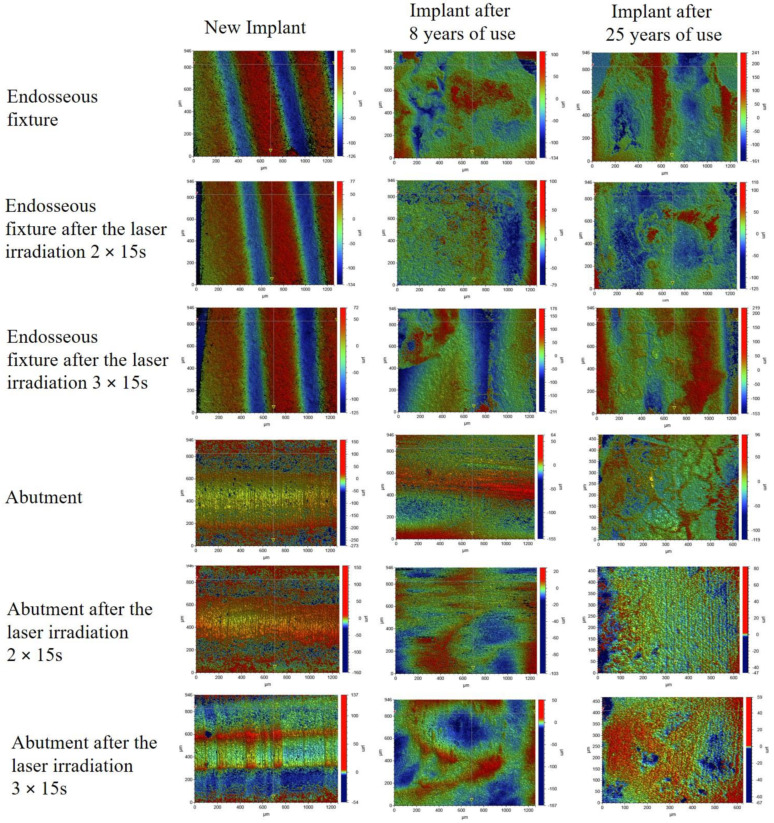
2D surface microgeometry maps generated by optical profilometry for the implant: new and those, removed after 8 and 25 years before and after the laser irradiation 25 W/15.000 Hz/10 µs, average = 3.84 W in the doses 2 × 15 s and 3 × 15 s.

In order to estimate the degradation of the implant surface after use by the patients, the roughness parameters of the implants were determined and compared with the new implant. The roughness parameters were also measured after the laser irradiation process in two doses: 2 × 15 s and 3 × 15 s. These tests were carried out in two ways in order to estimate the biocidal effectiveness of the laser and the surface changes caused by irradiation with the laser radiation. The test results are presented in Table 5 and Figure 6.

Surface microgeometry maps were generated by optical profilometry. The roughness parameters Ra, Rq, Rt were also determined from the mapped areas and the research results proved that abutment is a more representative area for roughness evaluation. The endosseous fixture areas are characterised by significant height differences. The research is not useless, however, because it confirms the significant accumulation of organic matter in the endosseous fixture areas. The organic matrix penetrating the thread cavities decreases the roughness parameters for this area. The parameter Ra = 38,471 for the new implant without laser modification decreased to Ra = 22,639 for the implant after 8 years of use and Ra = 37,034 for the implant after 25 years of use. These studies also show greater contamination of the endosseous fixture area for the implant after 8 years of use. The abutment surface is a much more representative area for the analysis of roughness parameters and the evaluation of laser performance. The research proved a significant decrease in the roughness parameters affected by laser irradiation for both the new implant and those used for 8 and 25 years. Moreover, following the research, the surface roughness depends on the applied laser dose. After using laser irradiation with the dose of 3 × 15 s, the smallest roughness parameters of the new implant and those of the implants used for 8 and 25 years were found.

## 5. Discussion

Dental implants are more and more often used in dentistry as a replacement for missing teeth, and, as a result, there has been a significant increase in the number of patients with symptoms of peri-implantitis (bone loss caused by peri-implant inflammation). One of the main causes of peri-implantitis is the lack of proper oral hygiene. In this case, undesirable biofilms containing a large number of microorganisms, including pathogens, develop on the implants. In this study, it was shown that the most abundant are Gram-negative bacteria, which was also confirmed by Kumar et al. [32]. Additionally, he found that the diversity of microorganisms accompanying peri-implantitis is smaller compared to healthy teeth. This phenomenon is undesirable because Gram-negative bacteria are more difficult to eradicate using pharmacological methods than Gram-positive bacteria.

The research allowed us to identify pathogenic and pyogenic species on the surface of the corroded dental implant, e.g., *Porphyromonas gingivalis, Prevotella intermedia, Treponema denticola, Tannerella forsythia* and *Fretibacterium fastidiosum*. Similar species that accompany peri-implantitis were found in studies by Zheng H. et al. and Sanz-Martin I, [33,34].

In this study, microorganisms were isolated from dental implants, which, after entering the bloodstream, show pathogenic potential in various parts of the body. Scientists have found that many of these species cause infections. *Dialister pneumosintes* causes diseases in the lungs, brain and root canals of the teeth. It is also isolated from pus and body fluids [35,36]. *Eggerthia catenaformis* occurs in dental abscesses [37]. *Eubacterium nodatum*, an opportunistic pathogen, is one of the dominant bacteria around implants [38]. *Prevotella nigrescens* plays a role in the pathogenesis of periodontal disease and by colonizing tissues it causes an overreaction of the immune system and increases the incidence of many diseases and infections [39]. *Streptococcus gordonii* can cause acute bacterial endocarditis when given systemic access. From the same genus, *Streptococcus salivarius* can cause iatrogenic meningitis [40]. The anaerobic *Tannerella forsythia* and *Treponema denticola*, which together with *Porphyromonas gingivalis* and *Tannerella forsythia* constitute the main harmful pathogens, cause chronic periodontitis [41].

The present research on the identification of microorganisms inhabiting titanium dental implants carried out by the MALDI TOF MS method for the breeding microorganisms and the NGS method for the breeding and non-breeding microorganisms confirmed the presence of many species of microorganisms with corrosive potential. The corrosive ability of microorganisms on dental implants is mainly due to the formation of multispecies biofilms and the mutual modulation of interspecies metabolism. Such relationships were confirmed by Periasamy and Kolenbrander for the microorganisms: *Porphyromonas gingivalis, Streptococcus oralis, Streptococcus gordonii, Actinomyces oris, Veillonella sp., Fusobacterium nucleatum, Aggregatibacter actinomycetemcomitans* [15]. Moreovers Al-Ahmad et al. demonstrated the corrosive properties and increased porosity of titanium dental implants by biofilms composed of the genera: Streptococcus, Veillonella and the species: *Fusobacteriaum nucleatum, Actinomyces naeslundii* [42]. Corte’s-Acha et al., among the species frequently coexisting in periodontopathogenesis affecting host cells and having corrosive effects on implants are: *Fusobacterium nucleatum* and *Porphyromonas gingivalis* [11]. The present research confirmed the presence of biofilm-forming and potentially corrosive bacteria of the genus *Streptococcus (S.anginosus, S.constellatus, S.gordonii, S. massiliensis, S. mutans, S.oralis, S. pneumonie, S. salivarius, S. sanguinis, S.sobrinus),* as well as the species of the genus *Veillonella (V.parvula, V.atypica)* and the species *Porphyromonas gingivalis.*

The type of material and the nature of the surface of dental implants (hydrophilic/hydrophobic) determine the number and biodiversity of microorganisms that inhabit them, which, as a result, can affect their susceptibility to corrosion [43]. The Zn-containing implant surfaces are characterised by a greater biodiversity of biofilms, including the species of microorganisms: *T. forsythia, P. nigrescens, S. sanguinis, P. aeruginosa, P. endodontalis, S. aureus, S. gallolyticus, S. mutans, S. parasanguinis, S. pneumoniae* and *C. albicans* compared to the Ti-containing implants. The Ti-containing implants showed less variability and a greater number of *Tannerella forsythia* and *Treponema denticola*.

The present studies confirmed the presence of *Tannerella forsythia* and *Treponema denticola*, as well as *Prevotella nigrescens* and other bacterial species not reported in the literature, capable of forming biofilms and characterised by corrosiveness (Prevotella, Bifidobacterium).

According to Rakic et al. the entire oral microbiome is capable of forming a biofilm, destroying the titanium-coated surfaces [44]. Pozhitkov et al. believe that the microbiome inhabiting the surfaces of dental implants contributes to microbiologically-induced corrosion due to electric potential generation. As a result, titanium is released from the implants [20]. The mechanisms of inducing titanium implants corrosion as a result of oxidation of the material surface and leaching of titanium into the environment have been known mainly for *Streptococcus mutans* [30]. This bacterium is able to acidify the environment and plays a significant role in the destruction of implants due to their discoloration, deformation of rough and smooth elements, pits and strong surface rusting. This species has also been detected in the current research.

Al-Ahmad et al. believe that corrosion susceptibility is mainly related to gene expression as an expression of adaptation to the microenvironment [42]. The viability and activity of biofilms may be due to the physical and chemical properties of the surface, which determine the metabolism of the microbes, but it may also be independent of the produced biofilm.

The biocorrosion of the titanium implant surface can contribute to an even faster inflammation process. The more effective the biofilm is, the rougher the implant surface. With high colonization by microorganisms, endotoxins are formed, stimulating inflammatory processes; therefore, effective methods of removing microorganisms are sought for the first stage of inflammation. Pharmacological methods are not always effective in this case. In addition, the use of effective antibacterial agents can develop antibiotic resistance.

Lasers are applied in various areas of life, including medicine and dentistry. They are increasingly used in clinical procedures during tissue treatment [45,46]. The research carried out so far focused mainly on the assessment of the influence of the laser used as a tool detection of caries and subgingival calculus, as a cutting tool, and, to a lesser extent, for root canal disinfection [47]. Recently the testing of lasers for surface decontamination in dentistry has also begun. This is to reduce the number of microorganisms, including pathogens that can contribute to inflammation. This method of decontamination is most often used in endodontics as an additional biocide by irradiating the canal walls [48]. In periodontics, the use of a laser as an adjunct did not lead to clinical improvement compared to conventional, non-surgical treatment alone [49]. Scientists have attempted to evaluate the effectiveness of laser treatment of peri-implant inflammation by comparing it with the conventional treatment. Some scientists did not notice any significant differences [50,51,52]. Other researchers proved that, in addition to surgical/non-surgical treatment of peri-implant mucositis and peri-implantitis, lasers have some clinical benefits [53]. Wawrzyk et al. showed that the laser used decontamination of the healing screws and the surrounding area shortened the time of wound healing [35]. It should be noted that the laser has a number of advantages: it does not cause antibiotic resistance, its use is not associated with a potential allergy/intolerance to biocidal components and it is a non-selective biocidal agent as it affects all biofilm microorganisms (with the efficiency of 88.05 to 100%). Medical diode laser reduces microorganisms depending on the surface they inhabit. In these studies, a reduction of 87.75–100% was achieved and a smaller reduction in the number of microorganisms of 38–100% was found on the dental composite [54]. On other cellulose material, Rybitwa et al. achieved 92.17–100.00% and on collagen material, 96–100% [55,56]. 

In addition, following from these studies, it does not have a negative effect on the implants (it neither changes the structure nor increases the roughness). The use of a laser can have an adverse effect on the surfaces to be illuminated when the dentist uses it in non-optimised doses, applying wrong techniques. 

To assess the effect of the laser on the material surface, the roughness value was examined with an optical profilometer and the surface structure was verified using confocal microscopy. Confocal microscopy allowed the assessment of organic matter contamination of the surface of dental implants in the area of Endosseous fixture and abutment. Endosseous fixture is characterised by a significantly greater amount of fluorescent material at blue laser light with the wavelength of λ = 408 nm. The area showing less fluorescence is abutment. These results correlate with the surface roughness of these implant fragments. The greater the surface roughness of a given implant component, the greater the surface area that fluoresces on the microscopic images.

Optical profilometry is a very commonly used research method that enables the characterisation of the materials surface. The non-contact three-dimensional measurement system using an optical profilometer will enable quick and accurate measurements of large areas. The basis of the optical profilometer operation is the phenomenon of light interference and the imaging of interference fringes. The monochromatic light beam, the source of which is the LED diode, is split in the interference lens. One part of the beam is reflected from the sample surface and the other part from the reference mirror. The returning beams interfere with each other, creating an interference pattern of the tested surface on the detector. The profile of this pattern and its location along the Z axis are the basis for mapping the surface microgeometry of the studied area in the 3D projection. Owing to the optical method, the measurements are very fast and take place without contact. This is an ideal solution for measuring depth profiles, roughness, as well as the extent and the level of material wear. This was applied in the research of new dental implants and those after being used for 8 and 25 years. After laser irradiation, the implants surface was also examined. The research was aimed at determining the roughness parameters and assessing the wear of the titanium material after the period of being used by patients. Surface changes were also observed as a result of surface irradiation with a laser. Particular attention was paid to the amplitude (height) parameters in relation to the reference plane, i.e., the arithmetic mean of the roughness—Ra, the mean square of the roughness—Rq, and the height of the largest profile cavity—Rt. The research results proved that abutment is the most representative area for the roughness parameters analysis. Surface smoothing was observed due to laser irradiation. No damaging effect of the laser on the tested surface of the implants was observed.

Excessive plaque deposition, which is not beneficial for dental implants, is favoured by an increase in the roughness of implant-prosthetic surfaces [57,58]. However, some researchers proved that there is no evidence that oral implant surfaces show a relationship between the biofilm development and the surface roughness [26,27].

The surface structure of the dental implants depends on the length of implant exposure to the direct action of external factors of the oral cavity, including microorganisms. If it is surrounded by bone all the time, it is less damaged and less corroded, as confirmed in this study.

## 6. Conclusions

1. Anaerobic bacteria dominate among the microorganisms inhabiting dental implants in patients with periimplantitis
2. Diode laser irradiation of the abutment surface and endosseouses fixture at optimised doses effectively reduces the number of microorganisms. Microbial reduction in abutment is greater than on endosseous fixture.
3. Properly selected doses of the diode laser effectively reduce microorganisms and they do not deteriorate the surface roughness of titanium implants.
4. The amount of corrosion of dental implants in patients with peri-implantitis is mainly influenced by the time of exposure to the environmental factors of the oral cavity, and to a lesser extent by the time of use.

## 7. Significance

Peri-implantitis is the process of bone loss around dental implants, most often caused by the adverse effects of microorganisms. This process is accompanied by pathogenic and corrosive microorganisms. The tested 810 nm diode laser, in optimised doses, can be used to reduce these microorganisms, and thus accelerate the treatment of peri-implantitis.

## Figures and Tables

**Table 1:** Primer used for amplification of 16S rRNA gene.

Primer Name	16S rRNA Region	Primer Sequence (5′-3′)
Bac341F	V3	CCTACGGGNGGCWGCAG
Bac806R	V4	GACTACHVGGGTATCTAATCC

**Table 2:** Microorganisms isolated from dental implants of the patients with full-blown peri-implantitis identified by the matrix-assisted laser desorption/ionization time-of-flight mass spectrometry (MALDI TOF MS) method.

Microorganisms	
Species of Bacteria	
Implant after 8 Years of Use (Patient 2)	Implant after 25 Years of Use (Patient 1)
*Citrobacter koseri*	*Carnobacterium divergens*
*Enterobacter cloacae*	*Klebsiella oxytoca*
*Erwinia perscinia*	*Lactococcus lactis*
*Lactococcus lactis*	*Serratia marcescens*
*Lactobacillus sakei*	*Staphylococcus capitis*
*Neisseria mucosa*	*Streptococcus constellatus*
*Staphylococcus capitis*	*Streptococcus massiliensis*
*Staphylococcus haemolyticus*	*Streptococcus sanguinis*
*Streptococcus constellatus*	
*Streptococcus pneumonie*	
*Streptococcus oralis*	
*Veillonella parvula*	
**Species of fungi**	
*Candida parapsilosis*	*Candida dubliniensis*
*Candida guilliermondii*	

**Table 3:** Microorganisms breeding and culture-depended under the laboratory conditions isolated from tooth implants of the patients with full-blown peri-implantitis identified to the species by the NGS method.

Microorganisms			
Gram-Positive Bacteria	Gram-Negative Bacteria	Anaerobic Bacteria	Bacteria with Corrosive Potential
*Bifidobacterium dentium*	*Anaeroglobus geminatus*	*Anaeroglobus geminatus*	*Bifidobacterium dentium*
*Bifidobacterium longum*	*Alloprevotella rava*	*Alloprevotella rava*	*Bifidobacterium longum*
*Clostridiales bacterium*	*Alloprevotella tannerae*	*Alloprevotella tannerae*	*Porphyromonas gingivalis*
*Denitrobacterium detoxificans*	*Capnocytophaga sputigena*	*Bifidobacterium dentium*	*Prevotella nigrescens*
*Eubacterium infirmum*	*Klebsiella oxytoca*	*Bifidobacterium longum*	*Streptococcus anginosus*
*Eubacterium brachy*	*Dialister pneumosintes*	*Clostridiales bacterium*	*Streptococcus constellatus*
*Eubacterium minutum*	*Neisseria oralis*	*Denitrobacterium detoxificans*	*Streptococcus gordonii*
*Eubacterium nodatum*	*Porphyromonas gingivalis*	*Dialister pneumosintes*	*Streptococcus mutans*
*Mogibacterium timidum*	*Prevotella denticola*	*Eubacterium infirmum*	*Streptococcus salivarius*
*Peptoniphilus lacrimalis*	*Prevotella nigrescens*	*Eubacterium brachy*	*Streptococcus sobrinus*
*Streptococcus anginosus*	*Prevotella pallens*	*Eubacterium minutum*	*Tannerella forsythia*
*Streptococcus constellatus*	*Prevotella baroniae*	*Eubacterium nodatum*	*Treponema denticola*
*Streptococcus gordonii*	*Prevotella genomosp*	*Mogibacterium timidum*	
*Streptococcus mutans*	*Prevotella melaninogenica*	*Peptoniphilus lacrimalis*	
*Streptococcus salivarius*	*Prevotella oralis*	*Phocaeicola abscessus*	
*Streptococcus sobrinus*	*Prevotella salivae*	*Porphyromonas gingivalis*	
	*Pyramidobacter piscolens*	*Prevotella denticola*	
	*Tannerella forsythia*	*Prevotella nigrescens*	
	*Treponema denticola*	*Tannerella forsythia*	
	*Treponema maltophilum*	*Treponema denticola*	
	*Treponema pectinovorum*	*Treponema maltophilum*	
	*Treponema socranskii*	*Treponema pectinovorum*	
	*Veillonella atypica*	*Treponema socranskii*	
		*Veillonella atypica*	

**Table 4:** Percent reduction in the number of pathogens isolated from the patients with peri-implantitis and from the ATTC collection after laser irradiation in 2 variants: 2 × 15 s, 3 × 15 s on the abutment and endosseous fixture 8- and 25-year-old dental implants.

Microorganisms		Type of Sample						
		Surface Irradiated	Irradiation Time					
			25 Years Old Implant			8 Years Old Implant		
			Unirradiated	2 × 15 s	3 × 15 s	Unirradiated	2 × 15 s	3 × 15 s
			Average Number of Microorganisms [CFU/mL]	Reduction [%]		Average Number of Microorganisms [CFU/mL]	Reduction [%]	
Gram-negative bacteria	*Klebsiella oxytoca*	Abutment	1.2 × 10^6^ ± 5.5 × 10^4^	100.00 *	100.00 *	1.4 × 10^6^ ± 3.5 × 10^4^	95.24 *	99.42 *
		Endosseous fixture		96.00 *	100.00 *		94.04 *	94.23 *
	*Escherichia coli* ATTC 25922	Abutment	1.9 × 10^6^ ± 5.7 × 10^5^	100.00 *	100.00 *	2.9 × 10^6^ ± 2.7 × 10^5^	98.87 *	99.00 *
		Endosseous fixture		100.00 *	100.00 *		93.10 *	98.00 *
Gram-positive bacteria	*Streptococcus constellatus*	Abutment	1.2 × 10^6^ ± 1.3 × 10^5^	100.00 *	100.00 *	1.1 × 10^6^ ± 1.3 × 10^4^	89.83 *	98.02 *
		Endosseous fixture		95.00 *	100.00 *		88.85 *	93.10 *
	*Staphylococcus aureus* ATTC 29213	Abutment	5.8 × 10^6^ ± 6.2 × 10^4^	100.00 *	100.00 *	4.6 × 10^6^ ± 2.2 × 10^4^	100.00 *	100.00 *
		Endosseous fixture		100.00 *	100.00 *		94.95 *	100.00 *
Fungi	*Candida guilliermondii*	Abutment	3.6 × 10^5^ ± 2.8 × 10^4^	100.00 *	100.00 *	2.6 × 10^6^ ± 2.6 × 10^3^	88.05 *	96.77 *
		Endosseous fixture		97.82 *	98.90 *		87.75 *	93.10 *
	*Candida albicans* ATTC 10231	Abutment	8.1 × 10^5^ ± 5.6 × 10^3^	100.00 *	100.00 *	6.1 × 10^6^ ± 5.6 × 10^3^	93.80 *	99.15 *
		Endosseous fixture		95.20 *	96.15 *		93.20 *	95.00 *

Mean ± standard deviation; * statistically significant difference versus the control samples; ANOVA and LSD at a significance level *p* < 0.05.

**Table 5:** Roughness parameters (the arithmetic mean—Ra, the square mean—Rq, and the height of the largest profile cavity—Rt) determined by optical profilometry for the implant: new, and those removed after 8 and 25 years before and after the 25 W/15.000 Hz laser irradiation/10 µs, average = 3.84 W in the doses of 2 × 15 s and 3 × 15 s.

Surface	New Implant	Implant after	Implant after
		8 Years of Use	25 Years of Use
Endosseous fixture	Ra = 38.471	Ra = 22.639	Ra = 37.034
	Rq = 45.842	Rq = 29.681	Rq = 240.811
	Rt = 210.589	Rt = 241.486	Rt = 401.369
Endosseous fixture after the laser irradiation	Ra = 40.532	Ra = 13.539	Ra = 16.411
2 × 15 s	Rq = 47.296	Rq = 101.438	Rq = 22.87
	Rt = 210.425	Rt = 180.654	Rt = 243.292
Endosseous fixture after the laser irradiation	Ra = 40.758	Ra = 30.477	Ra = 34.311
3 × 15 s	Rq = 47.176	Rq = 37.385	Rq = 43.103
	Rt = 197.443	Rt = 389.476	Rt = 371.761
Abutment	Ra = 12.673	Ra = 7.896	Ra = 15.76
	Rq = 19.781	Rq = 11.301	Rq = 20.285
	Rt = 430.766	Rt = 219.145	Rt = 215.504
Abutment after the laser irradiation 2 × 15 s	Ra = 7.315	Ra = 4.575	Ra = 0.838
	Rq = 10.032	Rq = 6.071	Rq = 82.387
	Rt = 316.254	Rt = 127.71	Rt = 129.033
Abutment after the laser irradiation 3 × 15 s	Ra = 2.313	Ra = 4.471	Ra = 0.691
	Rq = 4.826	Rq = 5.519	Rq = 58.673
	Rt = 190.445	Rt = 238.824	Rt = 125.867

## Data Availability

Not applicable.
